# Clinic, Ambulatory and Home Blood Pressure Monitoring for Metabolic Syndrome: Time to Change the Definition?

**DOI:** 10.3390/medicina61030434

**Published:** 2025-02-28

**Authors:** Christina Antza, Maria Sitmalidou, Andrej Belančić, Niki Katsiki, Vasilios Kotsis

**Affiliations:** 1 3rd Department of Internal Medicine, Aristotle University, Hypertension, Hypertension-24h Ambulatory Blood Pressure Monitoring Center, Papageorgiou Hospital, 56429 Thessaloniki, Greece; kris-antza@hotmail.com (C.A.); mariasitma@gmail.com (M.S.); 2 Department of Basic and Clinical Pharmacology and Toxicology, Faculty of Medicine, University of Rijeka, 51000 Rijeka, Croatia; a.belancic93@gmail.com; 3 Department of Nutritional Sciences and Dietetics, International Hellenic University, 57400 Thessaloniki, Greece; nikikatsiki@hotmail.com; 4 School of Medicine, European University Cyprus, Nicosia 2404, Cyprus

**Keywords:** metabolic syndrome, blood pressure, atherosclerotic cardiovascular disease risk, ambulatory BP, home BP, office BP, pulse wave velocity, early vascular aging

## Abstract

*Background and Objectives*: Metabolic syndrome (MetS) is considered a global epidemic, and its diagnosis is crucial, allowing early intervention and management. The main aim of this study was to examine any possible blood pressure (BP) differences based on office and out-of-office measurements in patients with and without MetS, and to investigate if any of these measurements correlated better with MetS. The secondary aim was to investigate any possible cardiovascular risk differences. *Materials and Methods*: The study population consisted of individuals attending the outpatient hypertension clinic. Office and out-of-office BP measurements were recorded in all of the patients, as well as different cardiovascular risk scores and echocardiography. MetS was defined according to ACC/AHA criteria. *Results*: A total of 282 (39.9% men) individuals (56.8 ± 15.8 years) were analyzed; 60.8% of them had MetS. The patients with MetS had a significantly higher systolic BP (SBP) in all of the BP measurements, higher ASCVD risk (22% vs. 12%), Framingham risk scores (11.8% vs. 6.9%), a significantly higher prevalence of LVH (49.2% vs. 22.7%) and early vascular aging (54.8% vs. 27.4%) compared with the patients without MetS (*p* < 0.05 for all). In a univariate analysis, MetS was significantly correlated with the average 24h SBP, daytime and nighttime ambulatory SBP, office SBP, and home SBP in the morning (*p* < 0.05). No significant differences were observed for any of the DBP measurements. Finally, 50.5% of the MetS patients had sustained hypertension, 15.2% masked hypertension, and 11.5% white-coat hypertension based on ABPM, and these values were 45.1%, 19.3%, and 13.6%, respectively, based on HBPM. Furthermore, most of the MetS patients had non-dipping hypertension (56.4%). *Conclusions*: The present findings highlight the importance of out-of-office BP measurements in the diagnosis of MetS, since both a high office and out-of-office SBP were significant features of the syndrome (whereas this was not the case with DBP). This is further supported by the increased prevalence of different hypertension phenotypes observed in the MetS patients. Higher ASCVD risk scores and LVH and EVA prevalence were also related to MetS, thus strongly supporting the necessity for early detection and treatment.

## 1. Introduction

Metabolic syndrome (MetS) is a cluster of conditions, including high blood pressure (BP), hyperglycemia, central obesity, and dyslipidemia, that altogether significantly increase the risk of cardiovascular disease (CVD), thus potentially leading to premature mortality [1,2]. The American Heart Association (AHA) considers MetS to be a global epidemic, affecting around one-quarter of people worldwide and being a significant cause of morbidity and mortality [3]. Therefore, the diagnosis of MetS is crucial, as it allows early intervention and management [4].

Hypertension is a critical component of MetS and is tightly connected with other risk factors, which collectively increase the CVD risk [3]. The accurate diagnosis and management of BP in individuals with MetS are pivotal in preventing long-term complications [5]. Historically, office BP measurement (OBPM) has been the cornerstone of hypertension diagnosis and monitoring [6]. However, evidence suggests that this approach has disadvantages, as OBPM alone cannot reveal two hypertensive phenotypes, i.e., masked and white-coat hypertension, nor can it recognize nocturnal dipping and morning surges [7]. Hence, as hypertension—based on OBPM—is one of the determinants of MetS criteria, there might be underdiagnosis of patients, who need to be evaluated with out-of-office BP measurements to ensure correct diagnosis and treatment [6,8,9,10].

The importance of out-of-office BP measurements is further highlighted by their correlation with CVD. A recent meta-analysis by our team showed that both ABPM and HBPM are positively associated with the CVD risk after adjusting for OBPM [11]. Furthermore, out-of-office BP measurements offer a better correlation as regards target organ damage, such as left ventricular hypertrophy, microalbuminuria, and arterial stiffness [12,13,14]. Besides the multiple measurements performed in a usual working day with this method, these correlations could be the result of recording BP alterations during the day and also the assessment of the dipping status. The presence of non-dipping status was associated with non-fatal CVD after a 20-year follow-up [15], while increased BP variability increases the risk of all-cause and cardiovascular mortality as well as coronary heart disease and stroke [16].

The aim of this study was to examine any possible differences in BP measurements (i.e., ABMP, HBPM, and OBPM) in patients with and without MetS and investigate if any of these measurements correlate better with the syndrome. Furthermore, we aimed to identify the rates of hypertension phenotypes (e.g., masked and white-coat hypertension) in patients with MetS. The secondary aim was to compare atherosclerotic CVD (ASCVD) risk scores, early vascular aging (EVA) [defined by carotid–femoral pulse wave velocity—cfPWV], and left ventricular hypertrophy (LVH) prevalence in patients with vs. without MetS.

## 2. Materials and Methods

### 2.1. Study Population

The study population consisted of 282 individuals who attended the Hypertension-24h ABPM Center of Excellence at the 3rd Department of Internal Medicine at Aristotle University of Thessaloniki in Greece. Participants were informed of the procedure and provided their written consent to participate in this study. Both a treated and untreated population was included in the study. We excluded patients aged <16 years as well as those who did not complete all BP measurements. Other exclusion criteria were previous history of CVD, secondary hypertension, end-stage renal disease, and concomitant systematic or inflammatory diseases. Furthermore, we did not include patients referred to our clinic due to high BP values (i.e., >150/90 mmHg) that needed further investigation or treatment modification.

### 2.2. BP Measurements

Three different types of BP monitoring (i.e., OBPM, HBPM, and 24h-ABPM) were performed in all participants, following the European Society of Hypertension (ESH) guidelines [6] and using validated devices [17]. Regarding OBPM, it was measured three times in the arm with the higher BP values, with the appropriate cuff size, by the same investigator (CA). The average of these three measurements was considered the “office BP”. The appropriate cuff size was considered any cuff with length 75–100% and width 35–50% of the arm circumference. Before measurement, the patient was seated comfortably for at least 5 min, without talking or drinking/eating, and measurements were made on both arms to identify the arm with the higher BP values. Hypertension based on OBPM was defined by BP measurements higher or equal to 140/90 mmHg.

Participants also underwent 24h-ABPM (Spacelabs 90217, Spacelabs Inc., Redmond, WA, USA) on a usual working day. The ABPM was placed in the non-dominant arm and measurements were performed every 30 min during the day and 60 min during the night, to ensure at least 70% valid BP recordings. Hypertension based on ABPM was defined by BP measurements higher or equal to 130/80 mmHg.

Furthermore, HBPM (WatchBP Home, Microlife AG Swiss Corporation, Widnau, Switzerland) was performed in all participants. Patients were informed of how to use the HBPM by the principal investigator (CA). The device had been set up to measure BP twice daily in a specific period of time. Two measurements were performed in the morning (06:00–09:00) and two in the evening (18:00–21:00), with an interval of 1 min. The BP measurements were conducted for 7 consecutive days. HBPM was considered as the average of the BP recordings, excluding the BP measurements from the first day. Hypertension based on HBPM was defined by BP measurements higher or equal to 135/85 mmHg.

### 2.3. ASCVD Risk Assessment

ASCVD risk assessment was conducted through detailed demographic, clinical, and laboratory evaluations. Baseline characteristics of the study population included age, sex, body mass index (BMI), and comorbidities such as hypertension, type 2 diabetes (T2D), hyperlipidemia, and MetS. Waist and hip circumferences were measured by the same investigator (CA) [18]. Self-reported smoking status and alcohol intake were recorded, whereas serum lipids, HbA1c, creatinine, and uric acid values were recorded based on medical records. Most of the participants performed the biochemical exams in the same laboratory (G.N Papageorgiou), whereas the rest preferred to perform them in private. Estimated glomerular filtration rate (eGFR) was calculated according to the CKD-EPI formula [19].

MetS was defined according to the American College of Cardiology (ACC)/AHA criteria [4]. Obesity was assessed using BMI, with patients categorized as underweight, normal weight, overweight, or obese according to the World Health Organization (WHO) criteria [20]. Central obesity was evaluated through waist and hip circumference measurements [21]. Hypertension and T2D were defined based on the guidelines by ESH and the American Diabetes Association (ADA), accordingly [6,22]. ASCVD risk was further quantified using the ASCVD and Framingham 10-year risk scores [21,23]. EVA was defined based on cfPWV measurements, following the recommendations of the ESH Working Group on Vascular Structure and Function [24,25]. Measurements were performed using the Complior System (Colson, Les Lilas, France) and according to standardized procedures, including supine positioning and a controlled laboratory temperature. cfPWV was calculated by dividing 80% of the distance between the carotid and femoral recording sites by the pulse transit time. Patients were classified as having EVA if their cfPWV exceeded age-adjusted reference values [26]. Finally, 2D echocardiography (Vivid E95, GE Healthcare, Chicago, IL, USA) was performed in all participants in order to record LVH. Left ventricular mass was calculated with the linear method (Cube formula), recommended by the American Society of Echocardiography/European Association of Cardiovascular Imaging (ASE/EACVI). Indexation of left ventricular mass to height raised to the allometric power of 2.7 (LVM/height^2.7^) was used over indexing to BSA, since it demonstrates better predictive value for cardiovascular outcomes and better detection of obesity-related LVH. Finally, in accordance with the ASE recommendations, reference upper limits of normal left ventricular mass by linear measurements are 95 g/m^2^ for female patients and 115 g/m^2^ for male patients [27].

### 2.4. Statistical Analysis

SPSS 25.0 (SPSS Inc., Chicago, IL, USA) was used to statistically analyze the data. Continuous variables are reported as mean ± SD and categorical variables as percentages. Normality was evaluated with the Kolmogorov–Smirnov or the Shapiro–Wilk test, as appropriate. MetS was analyzed as a categorical variable, dividing patients as those having MetS or not. Crosstabs and Chi-tests were performed to measure the differences between categorical variables. Logistic Regression Analysis was also performed to evaluate how different BP parameters may affect MetS prevalence. Statistical significance was defined as 2-sided *p* < 0.005.

## 3. Results

### 3.1. Descriptive Statistics

A total of 282 (39.9% men) individuals aged 56.8 ± 15.8 years were included in the present analysis. The baseline characteristics of the study population are summarized in Table 1, whereas their mean BP values based on office, home, and ambulatory measurements are presented in Table 2. Briefly, 60.8% of the study population had MetS and 37.2% had hypertension—30% were on a hypertension treatment—while 40.1% were normotensive based on the office and out-of-office BP measurements; 45.7% were obese, 17.5% had T2D, and 24.3% were current smokers.

### 3.2. Differences in BP Measurements and ASCVD Parameters Between Those with and Without MetS

Table 3 summarizes the comparisons of the different BP measurements between the patients with and without MetS. In details, the MetS patients had significantly higher SBP values compared with those without MetS in all of the BP measurements, i.e., mean 24h SBP (130 vs. 117 mmHg; *p* = 0.004), mean SPB daytime (132 vs. 120 mmHg; *p* = 0.008), mean SBP nighttime (119 vs. 111 mmHg; *p* = 0.007), home SBP (140 vs. 131 mmHg; *p* = 0.046), home SBP morning (138 vs. 135 mmHg; *p* = 0.003), home SBP evening (135 vs. 134 mmHg; *p* = 0.036), and office SBP (143 vs. 129 mmHg; *p* = 0.001). In contrast, none of the DBP measurements were significantly different between the two groups. Of note, regarding the heart rate (HR), only the mean HR nighttime and home HR morning measurements were significantly higher in the MetS patients (*p* = 0.047 for both comparisons).

Table 4 presents the differences in the ASCVD risk scores and the LVH and EVA prevalence in the patients with and without MetS. In brief, both the ASCVD and Framingham risk scores were significantly greater in the MetS patients (22 vs. 12%; *p* = 0.043 and 11.8 vs. 6.9%; *p* = 0.017, respectively). Furthermore, the prevalence of both LVH and EVA was significantly higher in the MetS patients (49.2 vs. 22.7%; *p* = 0.005 and 54.8 vs. 27.4%; *p* = 0.004, respectively).

### 3.3. Logistic Regression Analysis for Different BP Parameters and MetS

The MetS prevalence significantly correlated with the mean 24h SBP (*p* = 0.04), mean SBP daytime (*p* = 0.019), mean SBP nighttime (*p* = 0.018), home SBP morning (*p* = 0.005) and office SBP (*p* = 0.001), as shown in Table 5. In contrast, no significant correlations were observed for any of the DBP measurements.

### 3.4. Prevalence of BP Phenotypes Among Patients with and Without MetS

Among the MetS patients, 50.5% had sustained hypertension (vs. 28.2% in the patients without MetS, *p* < 0.05), 15.2% had masked hypertension (vs. 7.3% in the patients without MetS, *p* < 0.05) and 11.5% had white-coat hypertension (vs. 23.9% in the patients without MetS, *p* < 0.05). Therefore, 26.7% or 32.9% of the MetS patients presented with a hypertension phenotype that needed both office and ABPM or HBPM, respectively, for their evaluation (masked: 15.2% and white-coat: 11.5% for ABPM; masked: 19.3% and white-coat: 13.6% for HBPM).

Furthermore, only 27% of the MetS population had a normal dipping status (35.2% of the patients without MetS, *p* < 0.05), whereas the majority of these patients had non-dipping hypertension (56.4% vs. 42.1% of the patients without MetS, *p* < 0.05). Table 6 shows the prevalence of the BP phenotypes among the patients with MetS.

## 4. Discussion

BP is a main contributing factor of MetS, and, thus, detecting hypertension is of major importance to further evaluate patients and diagnose MetS. The present study revealed that not only office SBP but also out-of-office SBP correlated significantly with MetS, whereas DBP (either office or out-of-office measurements) had no correlation. Furthermore, a high percentage of the MetS patients presented with masked or white-coat hypertension, as well as non-dipping hypertension, all conditions that need out-of-office BP measurements to be diagnosed. Finally, the MetS patients had significantly higher ASCVD and Framingham risk scores, as well as higher rates of LVH and EVA.

The superior predictive value of out-of-office BP measurements, particularly in detecting hypertensive phenotypes like masked and white-coat hypertension, was attributed to its stronger association with CVD risk factors [28,29]. Furthermore, The Jackson Heart Study revealed that the prevalence ratio of masked hypertension was 1.38 for the patients with MetS compared to those without MetS (95% CI, 1.10–1.74), in almost 350 participants not receiving treatment for hypertension [30]. Regarding white-coat hypertension, the PAMELA study showed that the prevalence was 8.5% of the total population and the characteristics of this population were higher values of total cholesterol, serum triglycerides, and BMI and lower values of HDL cholesterol compared to those with normotension [31,32]. These results underscore the necessity of incorporating both ABPM and HBPM into clinical practice for the diagnosis of MetS as well as comprehensive CVD risk assessment in MetS patients, aligning with the present study’s emphasis on out-of-office BP monitoring.

Furthermore, the non-dipping pattern observed in 56.4% of the MetS patients in the present study warrants particular attention. The prevalence of the non-dipping status reported in our study is in accordance with previously published data. Hermida R et al. found out that the prevalence of a non-dipping profile was significantly higher among subjects with MetS (52.0% vs. 39.5% in subjects without MetS, *p* < 0.001) [33]. Tartan Z et al. reported an even higher percentage of non-dipping status in patients with MetS (61.4%) in 132 participants who underwent both OBPM and ABPM [34]. Non-dipping hypertension, where SBP fails to be appropriately reduced during sleep, is associated with more severe CVD outcomes, including greater risks of stroke and myocardial infarction [35]. This reinforces the need to expand the use of ABPM and HBPM in clinical practice, particularly to detect non-dipping patterns early on and establish uniform risk-based management strategies.

Furthermore, our findings underscored the role of systolic hypertension in the pathophysiology of MetS, particularly as it relates to CVD risk. The consistently elevated SBP values across all of the BP measurement modalities—office, home, and ambulatory—serves as an early indicator of increased CVD burden in MetS patients. This observation emphasizes the need for timely intervention to prevent long-term complications, such as CVD, by targeting SBP even at stages where it may not yet reach traditional diagnostic thresholds. Moreover, identifying patients with hypertension at earlier stages of MetS could provide an opportunity for personalized therapeutic approaches to mitigate CVD events.

Longitudinal research has further underscored the predictive power of baseline SBP and DBP levels for future MetS development. Furthermore, a 13-year prospective study in the RIVANA cohort demonstrated that a higher SBP measurement in MetS patients significantly increased the risk of adverse CVD outcomes [36]. The study analyzed data from 3976 participants over a median follow-up time of 12.8 years to investigate the association between MetS and CVD risk, mortality, and the premature onset of CVD events, quantified using rate advancement periods (RAPs) [36]. MetS was significantly associated with a 32% higher risk of major CVD events (HR 1.32; 95%CI: 1.01–1.74, *p* < 0.05) and an average advancement of 3.23 years (95%CI: 0.03–6.42, *p* < 0.05) for the occurrence of such events [36]. The CVD mortality risk also increased by 64% (HR 1.64; 95%CI: 1.03–2.60, *p* < 0.05) with a RAP of 3.73 years, while the all-cause mortality rose by 45% (HR 1.45; 95%CI: 1.17–1.80, *p* < 0.05) with a RAP of 3.24 years. Notably, the presence of each additional MetS component incrementally increased the risk of major CVD events by 22% (HR 1.22; 95%CI: 1.09–1.36, *p* < 0.05) with a RAP of 2.31 years (95%CI: 0.88–3.74, *p* < 0.05) [36]. These findings support the independent role of MetS and its components in amplifying CVD morbidity and mortality risks.

This raises the question of whether we should revisit hypertension thresholds, particularly for MetS patients, given their heightened CVD risk. Indeed, even SBP levels, traditionally considered within the ‘normal’ range, may indicate an increased risk for CVD in MetS populations. Personalized thresholds for BP in this group could improve risk stratification, allowing for more precise treatment strategies tailored to the unique risk profiles of MetS patients. Further research into these personalized thresholds is essential to refine hypertension management in this high-risk cohort.

The integration of advanced BP monitoring methods, such as ABPM and HBPM, into clinical practice offers significant advantages for CVD risk management in MetS patients. This approach complements traditional risk models, such as ASCVD and Framingham risk scores, by providing more accurate and real-time data on BP patterns. In the present study, we showed that MetS patients had significantly greater ASCVD and Framingham risk scores, and they were significantly more prone to LVH and EVA development. Therefore, incorporating measurements, such as CVD risk scores, LVH, and cfPWV, could enable better CVD risk prediction and more individualized therapeutic approaches in MetS patients. Of note, arterial stiffness has been associated with increased CVD risk [37].

The present study has certain strengths, including the performance of robust BP monitoring methods, a detailed CVD risk evaluation, the identification of hypertensive phenotypes, and the investigation of implications for redefining clinical practices. There are also limitations, including its cross-sectional design, single-center scope, inclusion of both treated and untreated hypertensive patients, lack of longitudinal data, and no randomization for the order of the BP measurements—it was based only on the preference of patients. Future studies should address these limitations.

## 5. Conclusions

The present study found that both high office and out-of-office SBP values were significant features of MetS (whereas this was not the case for DBP). The need for out-of-office BP measurements was also supported by the increased prevalence of different hypertension phenotypes (i.e., masked, white-coat, and non-dipping status) observed in the MetS patients. We suggest redefining BP measurement methods and propose a leading role of SBP to increase risk stratification and early detection in patients with MetS. Nevertheless, further research is necessary to refine the role of these BP measurements in high-risk populations (such as MetS patients), addressing limitations such as patient adherence and cost-effectiveness. Another important finding of the present study involves the increased ASCVD risk scores, as well as the higher LVH and EVA prevalence rates observed in the MetS patients, thus highlighting their raised CVD risk. These results strongly support the necessity for the early detection and treatment of the syndrome.

## Figures and Tables

**Table 1:** Baseline characteristics of the total study population.

	Mean	Standard Deviation
Age (years)	56.9	15.8
BMI (kg/m^2^)	30.4	5.8
HbA1c (%)	6.3	1.3
Total Cholesterol (mg/dL)	199	42
Triglycerides (mg/dL)	134	64
HDL cholesterol (mg/dL)	48	13
LDL cholesterol (mg/dL)	123	36
Uric Acid (mg/dL)	5.4	1.4
eGFR (mL/min/1.73 m^2^)	83.9	21.8
Waist (cm)	77	47
Hip (cm)	81	50
	Percentage, % (n)	
Sex (men)	39.9 (112)	
Type 2 diabetes	17.5 (49)	
Current smoker	24.3 (67)	
LV hypertrophy	43.7 (123)	
Hyperlipidemia	43.3 (122)	
BMI_WHO (categories)	Underweight: 0.4 (1) Normal: 15.2 (42) Overweight: 38.7 (109) Obese: 45.7 (130)	
EVA (early vascular aging)	54.8 (154)	
Metabolic syndrome	60.8 (171)	
Dipping status	Extreme dipping: 12.3 (35) Dipping: 27.0 (76) Non-dipping: 54.4 (154) Reverse: 6.3 (17)	
Blood pressure phenotype	Normotension: 40.1 (113) White-coat: 13.6 (38) Masked: 9.1 (26) Hypertension: 37.2 (105)	

BMI: body mass index; WHO: World Health Organization; HbA1c: glycated hemoglobin A1c; eGFR: estimated glomerular filtration rate; LDL: low-density lipoprotein; HDL: high-density lipoprotein; LV: left ventricular.

**Table 2:** Μean blood pressure values based on office, home, and ambulatory measurements in the total study population.

	Mean	Standard Deviation
Mean SBP 24 h (mmHg)	129	15
Mean DBP 24 h (mmHg)	77	11
Mean SBP day (mmHg)	132	15
Mean DBP day (mmHg)	79	11
Mean SBP night (mmHg)	122	16
Mean DBP night (mmHg)	71	11
Home SBP (mmHg)	141	16
Home DBP (mmHg)	86	12
Home SBP morning(mmHg)	139	16
Home DBP morning(mmHg)	84	12
Home SBP evening (mmHg)	139	18
Home DBP evening (mmHg)	83	12
Office SBP (mmHg)	140	17
Office DBP (mmHg)	84	12

SBP: systolic blood pressure; DBP: diastolic blood pressure.

**Table 3:** Differences in blood pressure measurements between patients with and without metabolic syndrome.

	Metabolic Syndrome	No Metabolic Syndrome	*p*
Mean SBP 24 h	130 (19)	117 (10)	0.004
Mean DBP 24 h	71 (16)	74 (7)	0.669
Mean SBP day	132 (24)	120 (4)	0.008
Mean DBP day	73 (18)	76 (10)	0.587
Mean SBP night	119 (16)	111 (25)	0.007
Mean DBP night	67 (16)	71 (10)	0.934
Home SBP	140 (19)	131 (15)	0.046
Home DBP	85 (14)	76 (16)	0.843
Home SBP morning	138 (33)	135 (21)	0.003
Home DBP morning	79 (19)	82 (15)	0.992
Home SBP evening	135 (29)	134 (13)	0.036
Home DBP evening	78 (20)	82 (12)	0.298
Office SBP	143 (24)	129 (26)	0.001
Office DBP	84 (18)	76 (16)	0.170

All values are expressed as mean (standard deviation) mmHg. SBP: systolic blood pressure; DBP: diastolic blood pressure.

**Table 4:** Atherosclerotic cardiovascular risk assessment and LVH and EVA prevalence in patients with and without metabolic syndrome.

	Metabolic Syndrome	No Metabolic Syndrome	*p*
Age (years)	60 (12)	56 (16)	0.05
Sex (men)	67.6%	32.4%	0.131
ASCVD 10-year risk score, mean (SD)	22 (13)	12 (10)	0.043
Framingham 10-year risk score, mean (SD)	11.8 (9)	6.9 (6)	0.017
LVH	49.2%	22.7%	0.005
EVA	54.8%	27.4%	0.004

ASCVD: atherosclerotic cardiovascular disease; LVH: left ventricular hypertrophy; EVA: early vascular aging; SD: standard deviation.

**Table 5:** Logistic Regression Analysis of blood pressure measurements and metabolic syndrome.

	OR	95%CI	*p* Value
Mean SBP 24 h	1.05	1.02–1.06	0.04
Mean DBP 24 h	0.82	0.80–1.38	0.606
Mean SBP day	1.04	1.04–1.05	0.019
Mean DBP day	0.75	0.70–1.01	0.534
Mean SBP night	1.05	1.01–1.06	0.018
Mean DBP night	0.89	0.85–1.25	0.906
Home SBP	1.02	0.99–1.03	0.171
Home DBP	1.01	0.83–1.25	0.843
Home SBP morning	1.04	1.03–1.05	0.005
Home DBP morning	1.01	1.00–1.08	0.872
Home SBP evening	0.96	0.90–1.10	0.123
Home DBP evening	0.93	0.89–1.12	0.241
Office SBP	1.03	1.02–1.05	0.001
Office DBP	0.95	0.92–1.10	0.432

OR: Odds Ratio; SBP: systolic blood pressure; DBP: diastolic blood pressure.

**Table 6:** Prevalence of different blood pressure phenotypes in patients with metabolic syndrome.

	Different Blood Pressure Phenotypes			
	During 24h measurement			
In patients with metabolic syndrome	Normotension	White-coat	Masked	Sustained hypertension
	22.8%	11.5%	15.2%	50.5%
	During Home Measurements			
	Normotension	White-coat	Masked	Sustained hypertension
	22%	19.3%	13.6%	45.1%
	Dipping status during night			
	Extreme dipping	Dipping	Non-dipping	Reverse
	10%	27%	56.4%	6.6%

## Data Availability

Data are contained within the article.

## Abbreviations

MetS	Metabolic syndrome
BP	Blood pressure
ASCVD	Atherosclerotic cardiovascular disease
OBPM	Office blood pressure measurement
ABPM	Ambulatory blood pressure measurement
HBPM	Home blood pressure measurement
PWV	Pulse wave velocity
EVA	Early vascular aging
HR	Heart rate
BMI	Body mass index
LVH	Left ventricular hypertrophy
HbA1c	Hemoglobin A1C
eGFR	Estimated glomerular filtration rate
LDL	Low-density lipoprotein
HDL	High-density lipoprotein
